# Evolution in lung IRI cell death research: a 20-year global bibliometric study (2005–2024)

**DOI:** 10.1097/MS9.0000000000004986

**Published:** 2026-06-15

**Authors:** Haoshuai Yang, Bojun Xu, Zihan Wang, Qi Chen, Xiaoyan Wang, Jin Zhang, Chaoyang Liang

**Affiliations:** a Department of Thoracic Surgery, China-Japan Friendship Hospital, Beijing, P.R. China; b Health Science Center, Peking University, Beijing, P.R. China

**Keywords:** Bibliometrix, ferroptosis, lung ischemia–reperfusion injury, regulated cell death, research hotspots

## Abstract

**Background:**

Lung ischemia–reperfusion injury (LIRI) is a central pathological mechanism underlying primary graft dysfunction after lung transplantation. Regulated cell death modalities – including apoptosis, ferroptosis, and pyroptosis – are increasingly recognized as key drivers of LIRI. However, research is fragmented, with shifting foci and limited integration of the overall knowledge structure.

**Methods:**

We retrieved 2462 English-language articles (2005–2024) from the Web of Science Core Collection and conducted a comprehensive bibliometric analysis using CiteSpace, VOSviewer, and Bibliometrix, examining publication trends, international collaborations, keyword co-occurrence, and co-citation networks.

**Results:**

The United States (941 publications) and China (809 publications) accounted for 71.1% of global output. Thematic evolution showed a transition from an early focus on “ischemia–reperfusion injury” and “inflammation” to mechanistic studies of “apoptosis” and “oxidative stress.” Since 2020, emerging pathways such as “ferroptosis,” “mitophagy,” and “exosomes” have become prominent, with “ferroptosis” surging in 2022–2023 as a leading frontier. Co-citation analyses revealed that U.S. and Chinese research ecosystems drive experimental modeling and theoretical innovation, respectively, while cross-regional collaboration remains limited.

**Conclusions:**

LIRI research is shifting from classical inflammation–apoptosis paradigms toward a multidimensional, mechanistically nuanced framework. The dual-core structure of China and the United States, coupled with increasing integration of basic and clinical studies, highlights a growing convergence across organ systems. The data-driven knowledge map provided here offers a strategic reference for future translational and therapeutic research.

## Introduction

Lung ischemia–reperfusion injury (LIRI) represents a severe and clinically consequential pathological process that arises in a broad spectrum of settings, including lung transplantation, cardiothoracic surgery with extracorporeal circulation, resuscitation from profound shock, and the restoration of acutely interrupted pulmonary blood flow^[^1,2^]^. Among these contexts, transplantation-associated LIRI has emerged as the most extensively investigated and clinically impactful paradigm, constituting a principal contributor to primary graft dysfunction (PGD) and perioperative mortality following lung transplantation^[^3,4^]^. Despite continuous advances in perioperative management and organ preservation strategies, the incidence and morbidity associated with LIRI remain unacceptably high, underscoring the fact that its underlying molecular determinants have yet to be comprehensively elucidated.

HIGHLIGHTS

- Bibliometric analysis of 20-year lung ischemia–reperfusion injury cell death research trends
- Shift from apoptosis to autophagy and ferroptosis as key focuses
- The United States and China lead global publication output and collaboration

In recent years, regulated mechanisms of cell death have gained prominence as a central theoretical framework for interpreting the initiation and progression of LIRI^[^5–7^]^. Beyond conventional necrosis and apoptosis, multiple forms of programmed cell death – including autophagy-related cell death, pyroptosis, ferroptosis, and necroptosis – have been shown to contribute to post-ischemic structural damage and inflammatory amplification within pulmonary tissue^[^8–11^]^. Importantly, these death modalities do not operate in isolation; rather, they are intricately intertwined with oxidative stress, mitochondrial dysfunction, and immune activation, and their relative dominance appears to vary across disease contexts and injury stages^[^10,12^]^. In the setting of lung transplantation–associated LIRI in particular, perturbations in iron homeostasis, lipid peroxidation, and inflammasome activation have been closely linked to specific cell death pathways, suggesting that the selective engagement of distinct death programs may directly shape graft functional outcomes^[^13^]^.

Notwithstanding the steady expansion of research on cell death mechanisms in LIRI, the field remains fragmented and insufficiently integrated. Current investigative efforts are disproportionately concentrated on emerging forms of regulated cell death, such as ferroptosis and pyroptosis, whereas other regulatory pathways that have been extensively characterized in ischemia–reperfusion injury of other organs remain comparatively underexplored in the pulmonary context. This imbalance reflects an immature and incompletely organized knowledge structure within the LIRI literature.

Bibliometric analysis offers a powerful means of delineating the evolution of research themes, the migration of scientific hotspots, and the reconfiguration of knowledge architectures at a macroscopic level. However, a systematic bibliometric assessment focused specifically on cell death mechanisms in LIRI is still lacking. To address this gap, the present study comprehensively retrieved relevant publications from 2005 to 2024 and applied CiteSpace, VOSviewer, and Bibliometrix to interrogate publication dynamics, collaboration networks, and cell death–related research hotspots. By constructing an integrated knowledge map of this field, we aim to provide a data-driven framework to inform future mechanistic investigations and translational research efforts.

## Materials and methods

### Data sources

Relevant literature for this study was retrieved from the Web of Science Core Collection (WoSCC). The search strategy was formulated as follows: TS1 = (“Cell Death” OR “Apoptosis” OR “Necroptosis” OR “Ferroptosis” OR “Autophagy” OR “Programmed Cell Death” OR “Caspase-mediated Cell Death” OR “Necrosis”) AND (“Lung” OR “Pulmonary” OR “Lung Injury” OR “Lung Ischemia”); TS2 = (“Reperfusion Injury” OR “Ischemia–Reperfusion Injury” OR “Ischemic Injury” OR “Reoxygenation Injury”); TS = TS1 AND TS2.

**Figure 1.**
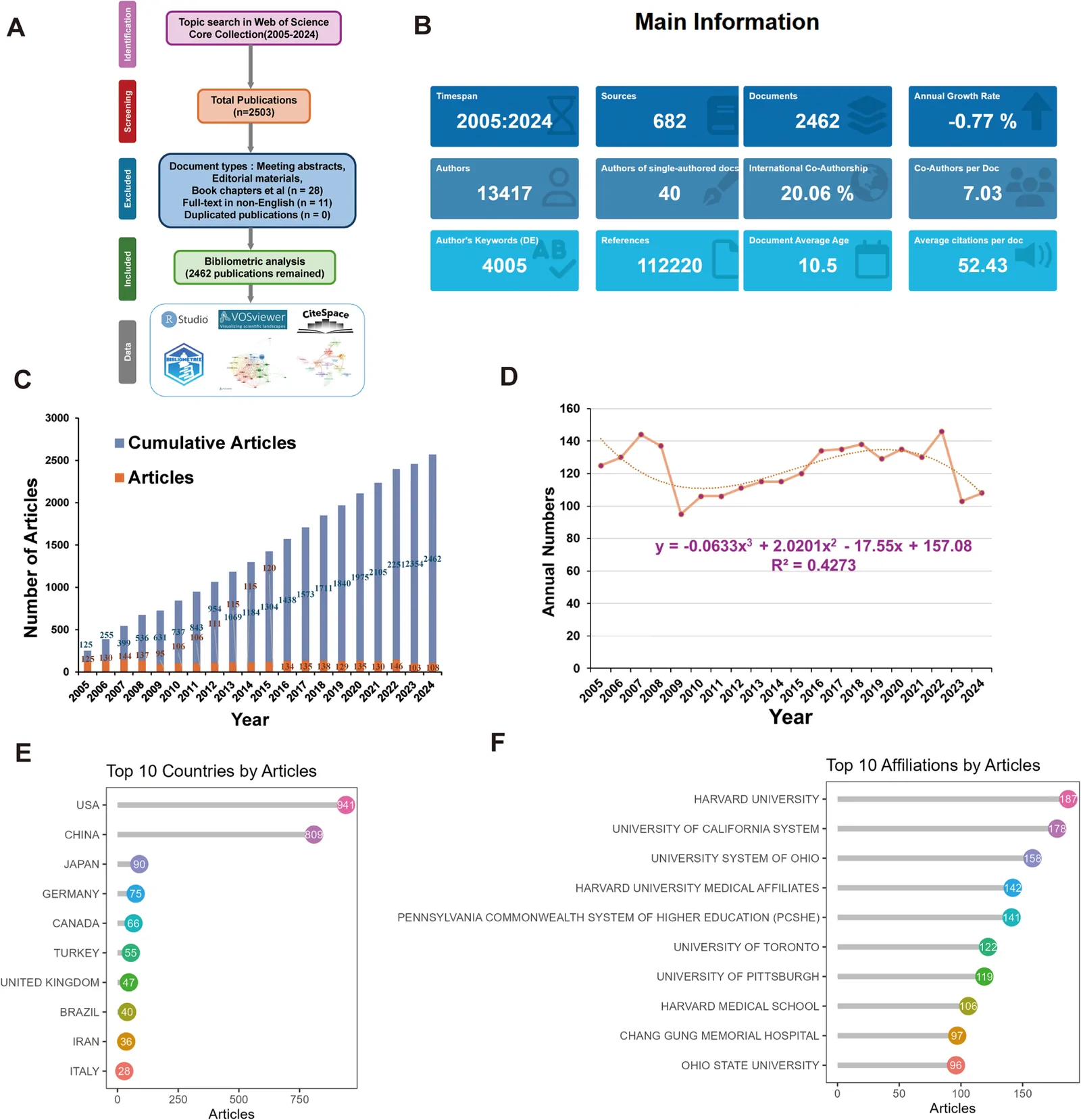
Literature screening and publication overview. (A) Flowchart of literature selection from the Web of Science Core Collection (2005–2024), showing inclusion and exclusion criteria. (B) Annual publication trends, cumulative publication counts, mean publication year, average citations per article, and proportion of international collaborations for the 2462 included publications. (C) Annual and cumulative publication counts for studies on cell death in lung ischemia–reperfusion injury (LIRI) from 2005 to 2024. (D) Polynomial fitting of annual publication trends, illustrating non-linear patterns and yearly fluctuations in research activity. (E) Top 10 contributing countries by publication count. (F) Top 10 contributing institutions by publication count.

The temporal scope was restricted to publications from 1 January 2005 to 31 December 2024. The initial search yielded 2503 records. After screening, only English-language articles and reviews were retained, and duplicate records were excluded. Ultimately, 2462 publications were included for subsequent bibliometric analyses and visualizations (Fig. 1A). The retained records comprised comprehensive metadata, including titles, authors, countries, institutions, journals, publication years, keywords, and cited references.

### Bibliometric analysis

The exported records were imported into multiple widely used bibliometric analysis platforms, including CiteSpace (version 6.4.R1), VOSviewer (version 1.6.20), and the Bibliometrix package (version 4.1.0) implemented in RStudio, to enable integrative visualization and quantitative assessment^[^14–16^]^. In addition, Microsoft Excel 2024 was used to generate descriptive bar charts and trend line graphs. These tools facilitated the extraction of key bibliographic information and the construction of visual knowledge maps, thereby providing a comprehensive depiction of the research landscape and its temporal evolution.

### Key parameter settings for knowledge mapping

To ensure the robustness and reproducibility of the bibliometric analyses, explicit parameter thresholds and algorithmic settings were applied across CiteSpace, VOSviewer, and Bibliometrix. In VOSviewer, networks were constructed using minimum threshold criteria, with link strength normalized by the “association strength” method, and clusters identified using a modularity-based community detection algorithm.

For CiteSpace, the data source was set to the WoSCC, with a time span of 2005–2024 and a 1-year time slice. Node selection criteria included g-index ≥ 25, LRF ≥ 2.5, L/N ≥ 10 for burst strength, and LBY ≥ 5 for burst duration. No pruning strategies were applied. Clustering was performed using the automatic clustering algorithm, and both modularity (Q) and silhouette values were calculated to evaluate cluster quality and structural validity.

In Bibliometrix, keyword co-occurrence networks were generated using an “automatic layout” algorithm, with community detection optimized via the Louvain algorithm to maximize modularity. Association strength normalization was applied, with the number of nodes set to 50. A repulsion force of 0.5 was used to regulate inter-node spacing, isolated nodes were removed, and a minimum co-occurrence threshold was imposed, such that each keyword was required to co-occur with at least two other keywords.

## Results

### Literature screening and publication overview

A systematic literature search was conducted using the WoSCC, covering the period from 1 January 2005 to 31 December 2024. The initial retrieval yielded 2503 records (Fig. 1A). Following predefined inclusion and exclusion criteria, non-research items such as conference abstracts, editorial materials, and book chapters (*n* = 28), non-English full-text publications (*n* = 11), and duplicate records (*n* = 0) were excluded. Ultimately, 2462 publications were retained for bibliometric analysis, all of which were English-language original articles or review papers.

These publications were distributed across 682 journals or conference proceedings and involved a total of 13 417 authors, with 40 papers authored by a single individual. After de-duplication, 4005 unique keywords were identified, and the included studies collectively cited 112 220 references. The compound annual growth rate (CAGR) of publications over the study period was −0.77%, indicating a modest overall downward trend when short-term fluctuations are smoothed (Fig. 1B). This metric was automatically calculated based on the annual publication counts from 2005 to 2024 using the standard formula: CAGR = (N_2024_/N_2005_)^1/T^ − 1, where T in the CAGR formula denotes the number of periods, equal to the end year minus the start year, and *N* represents the number of publications in a given year. In practice, however, annual output has exhibited pronounced variability since 2005, suggesting an unstable and non-linear evolution of research activity in this field.

The mean publication age of the included literature was 10.5 years. International collaborations accounted for 20.06% of all publications, with an average of 7.03 authors per article, reflecting a strong tendency toward collaborative research networks. Moreover, each publication received an average of 52.43 citations, underscoring the substantial academic impact and recognition of the field (Fig. 1B).

### Publication trends and geographic distribution

Annual publication output represents the most direct indicator of the rise and decline of a research topic. Between 2005 and 2024, studies addressing cell death in LIRI exhibited pronounced temporal fluctuations rather than a sustained upward trajectory (Fig. 1C). During the early phase (2005–2008), publication output increased rapidly, reaching 144 articles, followed by a plateau period from 2009 to 2015, during which annual outputs oscillated between 95 and 120 publications. Research activity intensified again after 2016, culminating in a recent peak in 2022 (146 articles), before declining in 2023 (103 articles) and 2024 (108 articles).

Polynomial fitting revealed a nonlinear temporal pattern in publication output; however, the modest explanatory power of the model (*R*^2^ = 0.4273) indicates that research activity in this field has been substantially influenced by multiple external factors, resulting in marked year-to-year variability (Fig. 1D).

From a geographic perspective, research output was highly concentrated in the United States (941 publications) and China (809 publications), which together accounted for 71.08% of the total literature. Japan, Germany, and Canada formed a second tier of contributing countries. A similarly pronounced concentration was observed at the institutional level. Harvard University ranked first with 187 publications, and its affiliated medical network – including Harvard Medical Affiliates (142 publications) – further consolidated its leading position. The University of California system (178 publications) and The Ohio State University system (156 publications) ranked second and third, respectively. Other prominent contributing institutions included the Pennsylvania State System of Higher Education (PCSHE), the University of Toronto, the University of Pittsburgh, and Chang Gung Memorial Hospital in Taiwan (Fig. 1F).

### Dual-map overlay analysis

**Figure 2.**
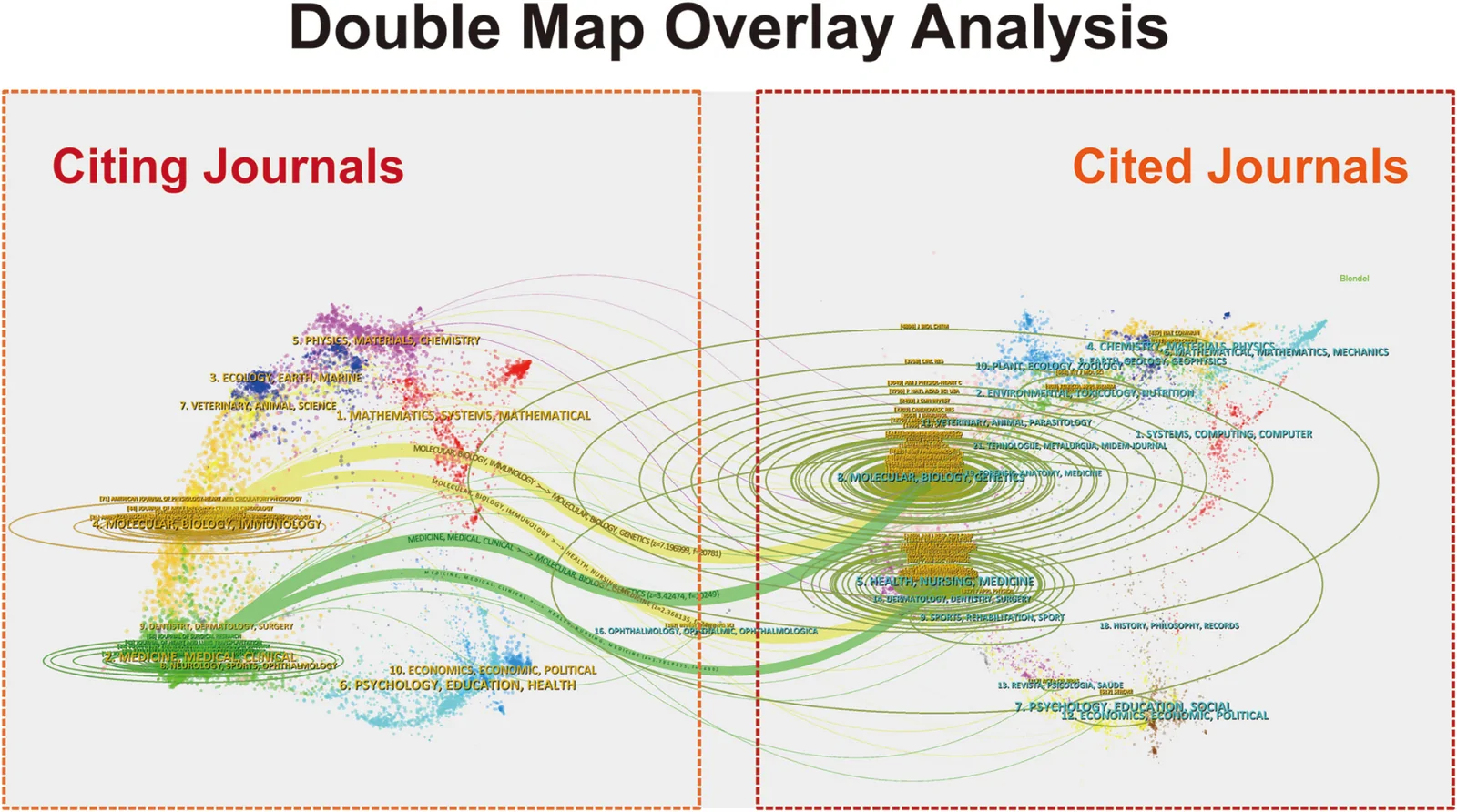
Dual-map overlay analysis.

The dual-map overlay visualization delineates the knowledge flow underpinning research on cell death in LIRI, with citing journals displayed on the left and cited journals on the right. As illustrated in Fig. 2, the citing journals were predominantly clustered within the domains of Medicine, Medical, Clinical, and Molecular Biology, Immunology, reflecting the strong clinical and mechanistic orientation of this research area. In contrast, the cited journals spanned a broad spectrum of disciplines, including Molecular Biology and Genetics, Health, Nursing and Medicine, and Environmental Toxicology and Nutrition. Notably, contributions from mathematics and computer science were also evident, underscoring the pronounced interdisciplinary nature of LIRI-related cell death research.

Displays knowledge flow between citing and cited journals, revealing disciplinary distribution and cross-disciplinary interactions in LIRI research.

We further examined self-citation behaviors among high-frequency citing and cited journals. The analysis revealed that the self-citation rates of all highly co-cited journals were below 13%, substantially lower than the 20% threshold defined by Clarivate as indicative of abnormal self-citation. Moreover, none of these journals were flagged for anomalous self-citation in the 2023 Journal Citation Reports (JCR). These findings substantiate the robustness and credibility of the identified knowledge dissemination pathways, indicating that they are not confounded by self-citation bias (Supplemental Digital Content Table S1, available at: http://links.lww.com/MS9/B154 and Supplemental Digital Content Table S2, available at: http://links.lww.com/MS9/B155).

Collectively, these results suggest that LIRI-related research is firmly anchored in high-quality journals and is propelled by dynamic interactions across basic science, clinical medicine, and pharmacology, thereby facilitating the discovery and translational application of emerging cell death mechanisms.

### Keyword analysis

**Figure 3.**
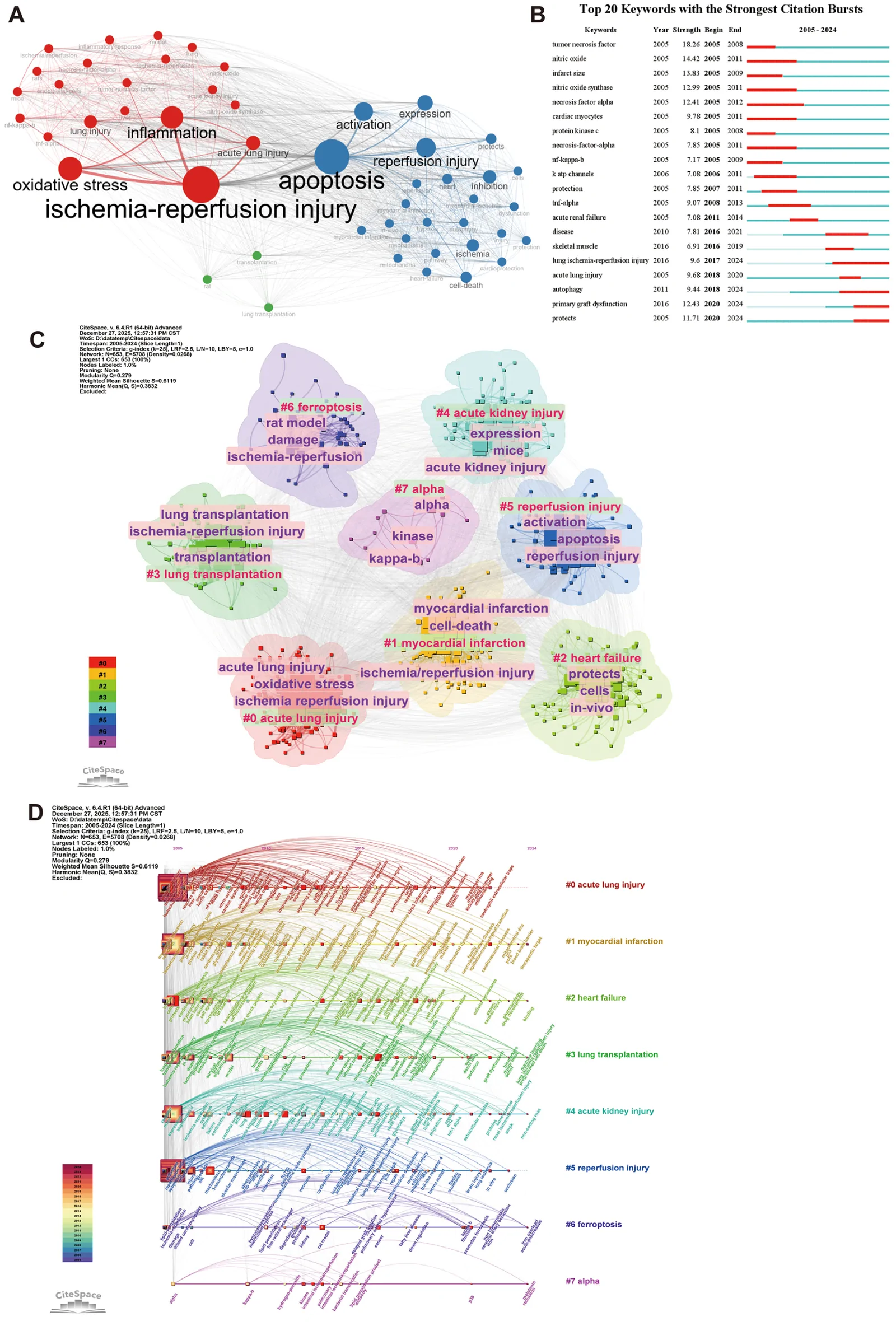
Keyword co-occurrence and thematic evolution. (A) Keyword co-occurrence network, with node size representing frequency and edge thickness indicating co-occurrence strength, highlighting core research themes. (B) Burst keyword analysis identifying emerging research hotspots over the past two decades. (C) CiteSpace clustering analysis, showing major knowledge clusters and thematic evolution paths. (D) Temporal evolution of keywords, illustrating dynamic changes in research focus and themes from 2005 to 2024.

Keyword co-occurrence network analysis elucidated the core research themes and underlying knowledge architecture of studies on LIRI (Fig. 3A). Nodes with high centrality – including ischemia–reperfusion injury, apoptosis, oxidative stress, and inflammation – constituted the foundational conceptual framework of the field. Among these, ischemia–reperfusion injury emerged as the most frequent and centrally positioned keyword, exhibiting dense connections with terms such as lung injury and acute lung injury. Together, these terms formed a prominent tissue injury–centered semantic cluster (red region), which underscores inflammatory responses, oxidative stress, and cell damage pathways during ischemia–reperfusion and represents the dominant paradigm of early research.

Concurrently, apoptosis occupied a central position within a distinct blue subnetwork, surrounded by terms including activation, mitochondria, and cell death, reflecting sustained and in-depth investigations into programmed cell death mechanisms. Notably, emerging modes of cell death, such as autophagy, although not yet embedded within the largest core cluster, have begun to establish significant connections with canonical concepts such as apoptosis and inflammation. Their gradual migration toward the network center suggests a rising prominence in elucidating LIRI pathogenesis. In addition, several peripheral nodes – such as transplantation, lung transplantation, and rat – were also evident, highlighting the continued importance of experimental models and clinical translational contexts as supporting pillars of this research domain.

Burst keyword analysis identified the most influential terms over the past two decades (Fig. 3B). Tumor necrosis factor (TNF-α; burst strength = 18.26) exhibited the earliest and most pronounced burst, spanning 2005 to 2008. Other terms, including necrosis factor alpha (strength = 12.41), kappa-B (strength = 7.17), and protection (strength = 7.85), showed concentrated bursts between 2005 and 2012, reflecting an early emphasis on inflammatory signaling pathways. Of particular note, autophagy (strength = 9.44) displayed a sustained burst beginning in 2018 and continuing through 2024, positioning it as a current research hotspot. This temporal shift indicates a gradual transition from classical apoptotic mechanisms toward alternative forms of regulated cell death.

CiteSpace-based clustering analysis further delineated seven major knowledge clusters, collectively outlining the intellectual evolution of LIRI research from traditional organ injury models toward emerging cell death mechanisms (Fig. 3C). Cluster #0 (acute lung injury) focused on experimental models and pathological features of acute lung injury, forming the cornerstone of lung-centered investigations. Clusters #1 (myocardial infarction) and #2 (heart failure) reflected sustained attention to cell death and functional decompensation in cardiac ischemia–reperfusion contexts. Cluster #3 (lung transplantation) centered on transplantation-related clinical scenarios, encompassing graft dysfunction and immunoregulatory mechanisms. Cluster #4 (acute kidney injury) highlighted the cross-organ commonalities of ischemia–reperfusion injury, whereas Cluster #5 (reperfusion injury) emphasized key signaling pathways activated upon reperfusion, such as NF-κB. Importantly, Cluster #6 (ferroptosis) emerged as a rapidly expanding hotspot in recent years, aggregating themes including rat model, damage, and ischemia–reperfusion. The inter-cluster links revealed strong associations between ferroptosis, lung transplantation, and acute lung injury, suggesting that ferroptosis may play a pivotal role in transplantation-associated LIRI and offering a promising entry point for future mechanistic studies and targeted interventions.

The thematic evolution map illustrated the dynamic temporal progression of keywords (Fig. 3D). During the early stage (2005–2010), research was dominated by terms such as acute lung injury, myocardial infarction, and heart failure. After 2011, autophagy, reperfusion injury, and apoptosis gradually gained prominence. Since 2020, emerging concepts – including ferroptosis, NLRP3 inflammasome, and pyroptosis – have appeared and formed independent trajectories, marking a paradigm shift toward diverse forms of regulated cell death.

Importantly, keyword co-occurrence analysis not only confirmed terms closely aligned with the predefined search strategy (e.g., cell death, apoptosis, ferroptosis), but also revealed several high-frequency keywords that were not explicitly included in the initial retrieval. Notably, NLRP3 inflammasome and pyroptosis, although absent from the search terms, exhibited significant burst trends and emerged as prominent hotspots after 2020 (Fig. 3B). This finding underscores the growing recognition of inflammasome activation and pyroptosis as critical contributors to LIRI pathophysiology, reflecting an expansion of research focus from classical apoptosis toward a broader spectrum of regulated cell death modalities. Furthermore, the rat model, frequently observed as a peripheral node, emphasizes the indispensable role of experimental animal models in mechanistic exploration, despite not constituting a core conceptual element of cell death itself.

### Analysis of collaborative networks among authors, countries, and institutions

**Figure 4.**
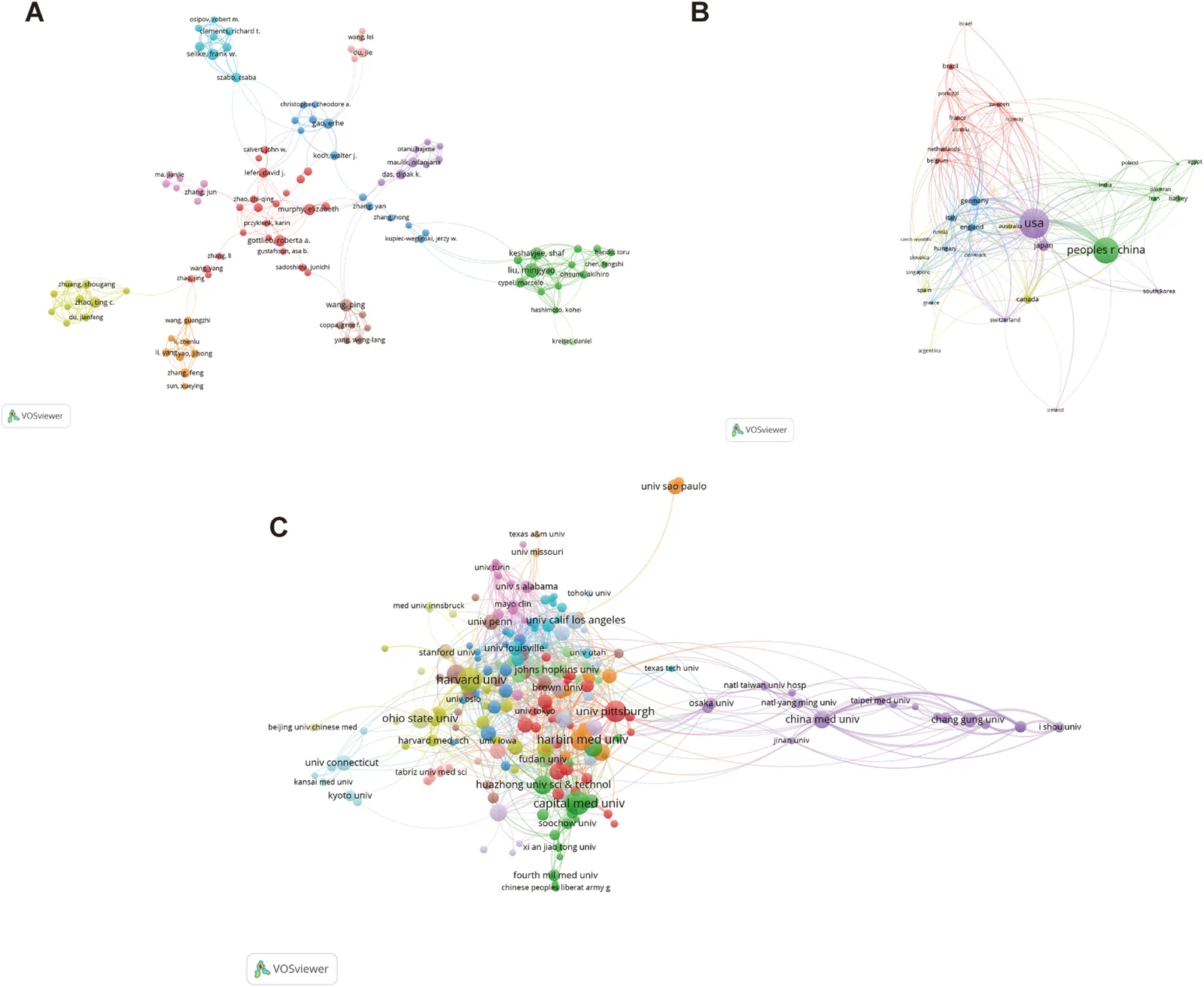
Collaboration networks among authors, countries, and institutions. (A) Author collaboration network, showing core research teams, intra-cluster connections, and intercontinental collaboration patterns. (B) Country-level collaboration network, highlighting bilateral collaborations between the United States and China, and contributions from other nations. (C) Institutional collaboration network, showing central hubs such as Harvard University, University of California system, University of Pittsburgh, and Chang Gung Memorial Hospital.

The author collaboration network delineates the cooperative structure and knowledge dissemination patterns among core research teams in the field of LIRI (Fig. 4A). A distinct and tightly interconnected cluster dominated by Chinese scholars – represented by Zhang, L., Wang, J., and Chen, Y. – was identified. This group exhibits frequent intra-cluster collaboration but maintains relatively limited direct connections with international research teams. Their work predominantly centers on themes such as lung transplantation, rat models, and inflammatory responses, reflecting a strong emphasis on experimental models and methodological frameworks^[^17–22^]^. In parallel, a large collaborative network led by U.S.-based researchers, including Koch, J., Murphy, E., and Gottlieb, R.A., was observed. This network – largely affiliated with Harvard University, the University of Pennsylvania, and The Ohio State University system – focuses on mechanistic investigations into apoptosis, mitochondrial dysfunction, and cell death pathways, underscoring a robust foundation in molecular biology and pathophysiology^[^23–26^]^.

Notably, smaller clusters involving authors such as Huang, X., and Zhou, Q., exhibited minimal connectivity with other groups, forming relatively isolated collaboration patterns that have yet to be fully integrated into the mainstream research network^[^26,27^]^. Researchers from Japan (e.g., Keshavjee, S.; Hashimoto, K.), Europe (e.g., Szabo, C.; Clements, R.T.), and Canada (e.g., Calvert, J.W.) demonstrated localized collaborations; however, these networks remained comparatively fragmented, with limited evidence of large-scale intercontinental integration. In addition, author-level academic contributions were quantified and ranked using the H-index (Table 1).

**Table 1:** Leading authors and academic metrics.

Author	h_index	g_index	m_index	TC	NP	PY_start
Liu Mingyao	17	23	0.85	1023	23	2006
Gottlieb Roberta A.	15	15	0.75	2030	15	2006
Murphy Elizabeth	15	15	0.75	2797	15	2006
Wang Ping	15	17	0.789	658	17	2007
Kron Irving L.	14	15	0.7	834	15	2006
Laubach Victor E.	14	16	0.7	1030	16	2006
Yip Hon-Kan	14	15	1.077	737	15	2013
Keshavjee Shaf	13	17	1.083	560	17	2014
Gao Erhe	12	12	0.6	1029	12	2006
Nakao Atsunori	12	12	0.6	842	12	2006

Lists the top contributing authors in this research field, ranked by H-index, total publications, and citation impact, reflecting their scholarly contributions to LIRI research.

The country-level collaboration network illustrates the geographic distribution of global research capacity and cooperative patterns (Fig. 4B). The United States and the People’s Republic of China emerged as the two most prolific contributors, with a prominent collaborative link between them (highlighted by red edges), indicating substantive bilateral engagement in LIRI research. Other developed countries, including Japan, Germany, and Canada, formed close collaborative ties with the United States, whereas emerging research nations, such as Turkey, Iran, and Brazil, primarily participated through unidirectional citation flows or limited cooperative efforts. Notably, the collaboration intensity between China and the United States was the strongest, reflecting a relatively stable and sustained bilateral partnership within the global research landscape.

Institutional collaboration analysis further refined these cooperative patterns by identifying specific organizational units (Fig. 4C). Harvard University, the University of California system, the University of Pittsburgh, and Chang Gung Memorial Hospital constituted a central collaborative hub, characterized by frequent co-authorship and sustained academic exchange. Several Chinese institutions – including Capital Medical University, Fudan University, and Tongji Medical College of Huazhong University of Science and Technology – also formed robust internal collaboration networks; however, their direct engagement with leading international institutions remained comparatively limited.

Harvard Medical School and its affiliated hospitals represent globally renowned centers for both clinical and basic biomedical research, with long-standing expertise in cardiovascular biology and ischemia–reperfusion injury. Their seminal contributions to mitochondrial function and cell death signaling pathways have been widely cited and form a cornerstone of contemporary cardiopulmonary protection paradigms. Mechanistic research teams in the United States and Europe, exemplified by the work of Gottlieb and colleagues, have made substantial advances in mitochondrial autophagy and signal transduction regulation^[^26^]^. In Canada, the Toronto Lung Transplant Program – led by Shaf Keshavjee – occupies a leading international position in clinical intervention strategies, organ preservation, and the development of *ex vivo* lung perfusion (EVLP) technologies for transplantation-related ischemia–reperfusion injury. Numerous innovations from this group have been successfully translated into routine clinical lung transplantation practice^[^28–30^]^.

Other institutions, such as Chang Gung Memorial Hospital in Taiwan and the University of Pittsburgh Medical Center, have also contributed to experimental modeling and multi-omics investigations in lung transplantation and ischemia–reperfusion injury. However, based on the current publicly available literature, these centers have yet to establish a level of global mechanistic influence and clinical translational impact comparable to that of the Toronto Lung Transplant Program.

### Multiple Correspondence Analysis and co-citation network analysis

**Figure 5.**
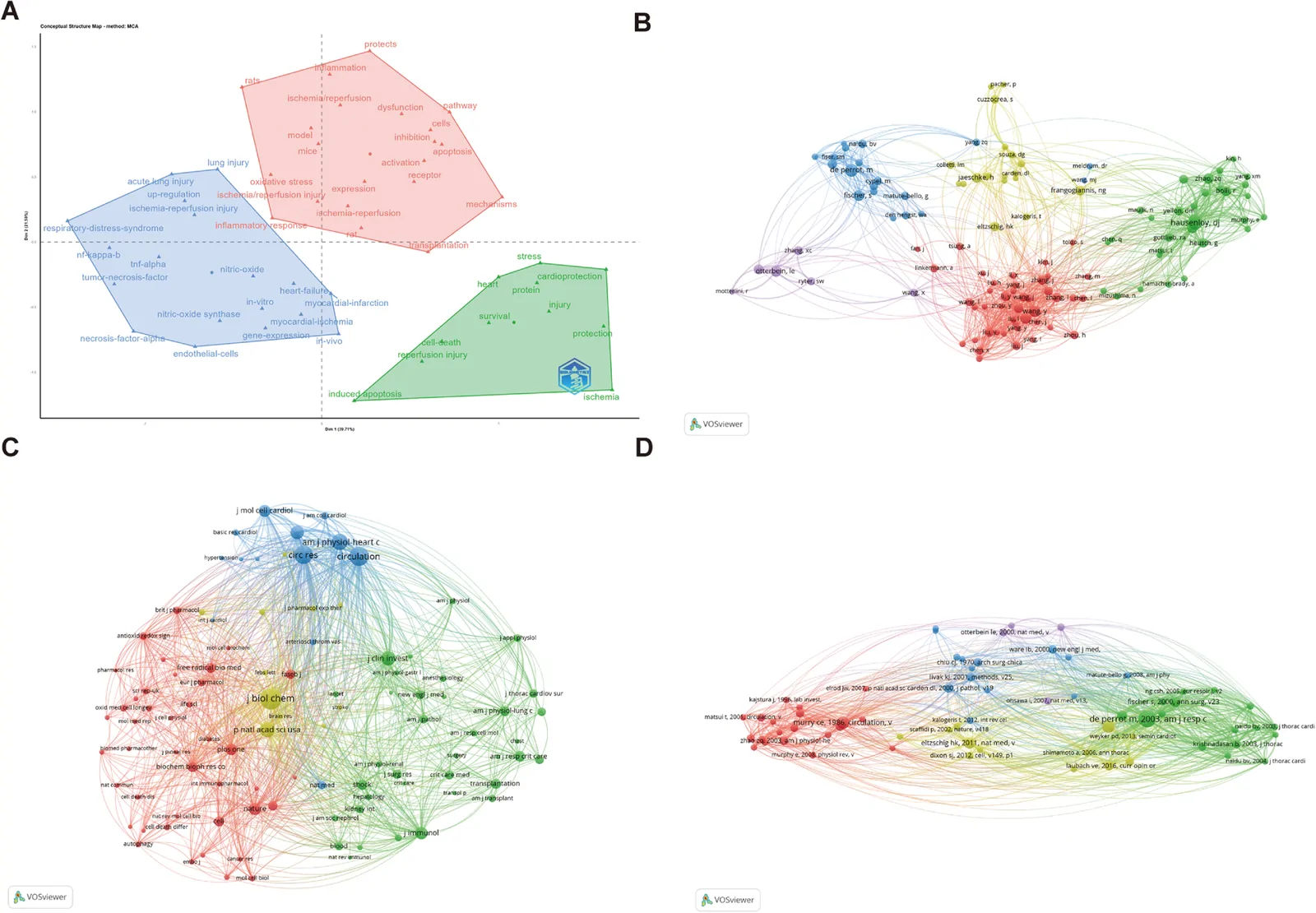
Multiple correspondence analysis (MCA) and co-citation networks. (A) MCA plot displaying three primary semantic clusters representing traditional inflammation – apoptosis, oxidative stress – endothelial dysfunction, and protective intervention research themes. (B) Author co-citation network revealing influential researchers and collaborative clusters. (C) Journal co-citation network identifying thematic clusters of high-impact journals and their disciplinary distribution. (D) Document co-citation network showing core publications in LIRI research and their intellectual lineage.

Multiple correspondence analysis (MCA) and co-citation network analysis were employed to elucidate the evolution of knowledge structures and core academic trajectories in the field of LIRI. In the MCA map (Fig. 5A), keywords were grouped into three major semantic clusters. The red cluster, centered on inflammation, apoptosis, and ischemia–reperfusion injury, represents the traditional inflammation–apoptosis research axis. The blue cluster emphasizes acute lung injury, oxidative stress, nitric oxide, and endothelial cells, reflecting pathophysiological processes related to oxidative stress and endothelial dysfunction. The green cluster, composed of ischemia, cardioprotection, protection, and survival, points toward research focused on protective intervention strategies.

The author co-citation network revealed the knowledge structure and influence of high-impact researchers in the LIRI field (Fig. 5B), forming multiple well-differentiated clusters. The red cluster, represented by Chinese scholars Zhang, J., Wang, Y., Chen, J., and Liu, Y., focuses on LIRI model construction, inflammation regulation, and oxidative stress mechanisms, establishing a mature localized research paradigm^[^31–34^]^. The green cluster, dominated by transatlantic collaborations, includes Hausenloy, D.J., Murphy, E., Gottlieb, R.A., Zhao, Z.Q., and Yang, X.M., with sustained attention to apoptotic signaling, mitochondrial dysfunction, and reperfusion injury salvage kinase (RISK) pathways, forming the foundational theory of cell death and cardiopulmonary protection^[^23,35^]^. The yellow cluster, represented by Jaeschke, H., Souza, D.G., Carden, D.L., and Cuzzocrea, S., centers on neutrophil activation following ischemia–reperfusion, bridging fundamental mechanisms^[^36–38^]^. The blue cluster, comprising De Perrot, M., Fischer, S., and Cyplen, M., from Switzerland, Germany, and Canada, emphasizes clinical trial research^[^28,39^]^. A small peripheral purple cluster, represented by Otterbein, L.E., and Matterlini, R., focuses on carbon monoxide and gas-mediated protective interventions; though limited in scale, it demonstrates potential for future growth^[^40^]^.

The journal co-citation network revealed the intellectual landscape and interdisciplinary integration of high-impact journals in LIRI research (Fig. 5C, Table 2). It formed four main thematic clusters: the red cluster (Nature, Cell, Molecular Cell Biology, Autophagy, Cell Death & Differentiation) underpins molecular mechanism studies, including apoptosis, autophagy, and ferroptosis; the green cluster (Transplantation, Am J Respir Crit Care Med, Critical Care Medicine, Chest) centers on lung transplantation, ARDS, and perioperative/ICU care, bridging basic and clinical research; the blue cluster (Circulation, J Am Coll Cardiol, Am J Physiol–Heart Circ Physiol) highlights shared mechanisms across organ systems, particularly cardiopulmonary interactions, mitochondrial dysfunction, and inflammation; the yellow cluster (Free Radical Biology & Medicine, Antioxidants & Redox Signaling, British Journal of Pharmacology) emphasizes oxidative stress and pharmacological interventions. JBC and PNAS occupy central hub positions, connecting clusters and integrating knowledge, while PLOS ONE facilitates cross-cluster dissemination, and review journals such as Nature Reviews Molecular Cell Biology and Cell Death & Disease drive conceptual synthesis and paradigm evolution.

**Table 2:** Highly cited articles and citation impact.

Paper	DOI	Total Citations	TC per Year	Normalized TC
Szabo C, 2007, Nat Rev Drug Discov	10.1038/nrd2222	1704	89.68	15.09
Kalogeris T, 2012, Int Rev Cel Mol Bio	10.1016/B978-0-12-394 309-5.00006-7	1632	116.57	24.57
Prabhu SD, 2016, Circ Res	10.1161/CIRCRESAHA.116.303577	1615	161.50	30.20
Matute-Bello G, 2008, Am J Physiol-Lung C	10.1152/ajplung.00010.2008	1400	77.78	15.54
Motterlini R, 2010, Nat Rev Drug Discov	10.1038/nrd3228	1376	86.00	20.27
Andersson U, 2011, Annu Rev Immunol	10.1146/annurev-immunol-030409-101 323	1191	79.40	17.43
Murphy E, 2008, Physiol Rev	10.1152/physrev.00024.2007	1188	66.00	13.19
Ryter SW, 2007, Antioxid Redox Sign	10.1089/ars.2007.9.49	1040	54.74	9.21
Elrod JW, 2007, P Natl Acad Sci USA	10.1073/pnas.0705891104	975	51.32	8.63
Spinale FG, 2007, Physiol Rev	10.1152/physrev.00012.2007	900	47.37	7.97

Lists the top 10 most cited articles globally in this research field, including total citations and normalized citation counts, illustrating high-impact research outcomes.

The document co-citation network elucidated the knowledge structure and theoretical lineage of high-impact publications in LIRI (Fig. 5D). The network exhibited a highly interconnected, multi-centered distribution, tracing knowledge evolution from basic mechanisms to clinical translation. Five core clusters were identified. The red cluster, represented by Murry, C.E. (1986, Circulation), established classical ischemia–reperfusion mechanisms and the concept of ischemic preconditioning^[^41^]^. The green cluster, represented by De Perrot, M. (2003, Am J Respir Crit Care Med), Naidu, B.V. (2003/2004, J Thorac Cardiovasc Surg), and Krishnadasan, B. (2003), focused on lung transplantation–related ischemia–reperfusion and organ protection strategies, facilitating translation from bench to bedside^[^42–44^]^. The blue cluster, with Otterbein, L.E. (2000, Nat Med), Carden, D.L. (2000, J Pathol), and Eltzschig, H.K. (2011, Nat Med), investigated molecular mechanisms including polyamine-albumin PNA, adenosine, and HIF-1α, elucidating inflammation, mitochondrial dysfunction, and cell death pathways^[^45–48^]^. The yellow cluster, represented by Kalogeris, T. (2012, Int Rev Cell Mol Biol), Scaffidi, P. (2002, Nature), and Dixon, S.J. (2012, Cell), encompassed diverse necrotic pathways in cellular biology^[^49–51^]^. Additionally, Elrod, J.W. (2007, PNAS) and Ohsawa, I. (2007, Nat Med) occupied intersecting positions across clusters, demonstrating strong cross-disciplinary influence and facilitating knowledge integration across organ systems^[^52,53^]^.

### Thematic evolution and trends

**Figure 6.**
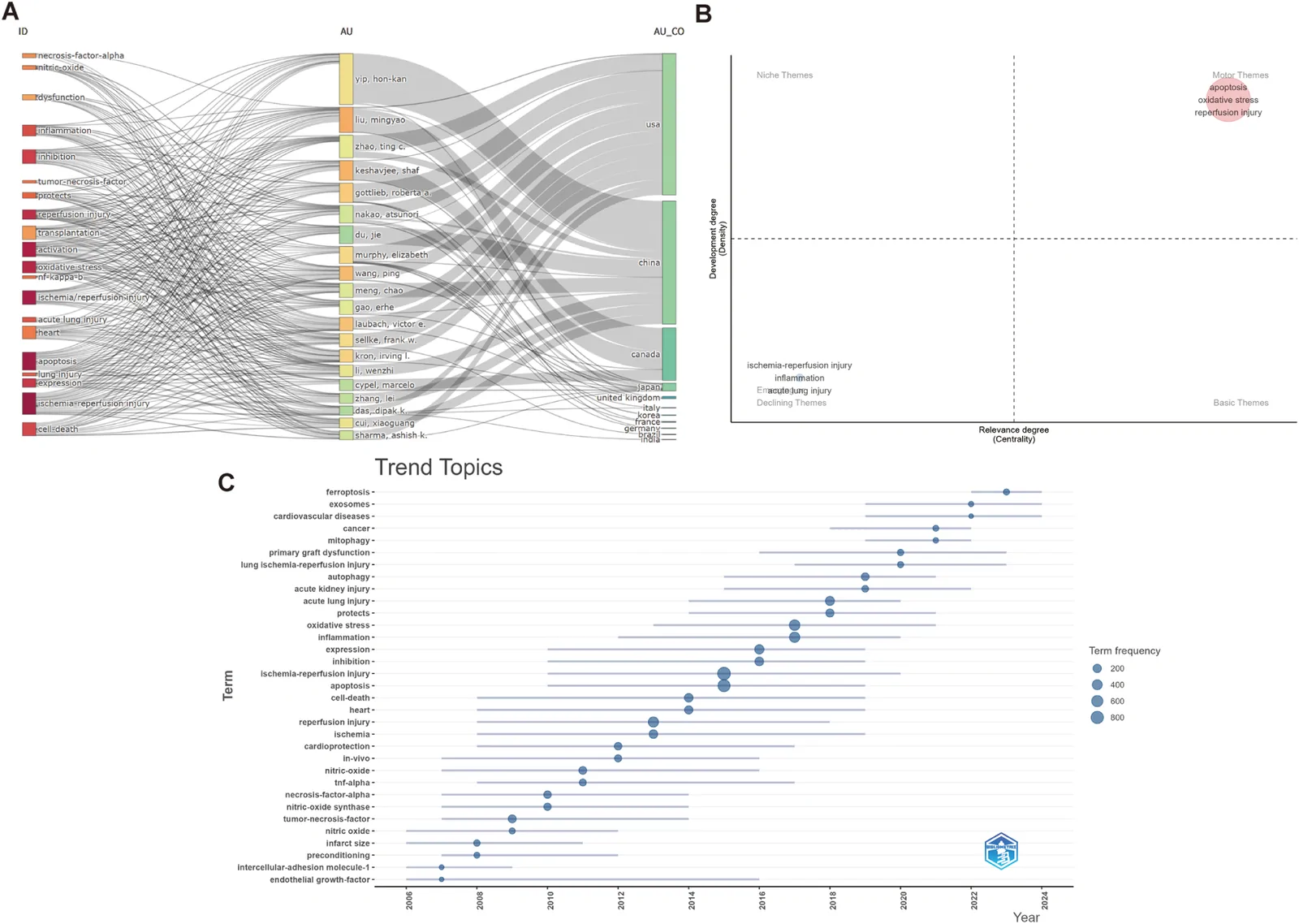
Thematic evolution and research trends. (A) Keyword–author–country Sankey diagram, illustrating multidimensional interactions among research topics, leading authors, and contributing countries. (B) Thematic centrality analysis, plotting research topics by relevance (centrality) and development (density), highlighting core driving themes and relatively declining topics. (C) Temporal evolution of trending keywords from 2005 to 2024, reflecting ongoing clinical and cross-disciplinary research hotspots.

The keyword–author–country Sankey diagram illustrates multidimensional interactions among core concepts, leading researchers, and contributing nations in LIRI research (Fig. 6A). The United States, China, and Canada dominate the field: the United States leads in apoptosis, reperfusion injury, inflammation, and cell death (e.g., Murphy, E.; Gottlieb, R.A.); China in ischemia–reperfusion injury, oxidative stress, lung injury, and transplantation (e.g., Liu, M.; Zhang, L.; Zhao, T.C.); and Canada in activation, inhibition, and cardiopulmonary signaling (e.g., Du, J.; Laubach, V.E.). Emerging topics such as ferroptosis, mitophagy, and exosomes have grown rapidly since 2020, driven by collaborations among Chinese, U.S., and select European teams, forming new international research hotspots. Keywords like necrosis-factor-alpha, tumor-necrosis-factor, and NF-kappa-B demonstrate cross-regional connectivity, whereas clinically oriented terms such as transplantation and acute lung injury cluster mainly in the United States, China, and Canada, reflecting shared translational priorities.

Thematic centrality analysis positions research topics along relevance (centrality) and development (density) (Fig. 6B). Motor themes – apoptosis, oxidative stress, and reperfusion injury – exhibit high centrality and development, representing core mechanistic nodes driving LIRI research. Declining themes – including ischemia–reperfusion injury, inflammation, and acute lung injury – show reduced centrality and density, indicating a shift from broad pathological descriptions toward a molecular-level mechanistic focus.

Temporal trend analysis traces LIRI research from 2005 to 2024 (Fig. 6C). Early studies (2006–2010) emphasized endothelial growth factor, preconditioning, and infarct size, reflecting vascular and tissue damage assessment. Mid-phase research (2010–2016) centered on apoptosis, reperfusion injury, oxidative stress, and inflammation, marking a move toward cell death mechanisms. From 2016 onward, autophagy rose in prominence, while mitophagy, exosomes, and ferroptosis expanded rapidly, with ferroptosis peaking in 2022–2023. Clinical terms such as lung ischemia–reperfusion injury and PGD remained active, and cross-disciplinary keywords – including cardiovascular diseases, cancer, and acute kidney injury – emerged, highlighting the translational progression toward lung transplantation, organ protection, and multi-organ mechanistic studies.

## Discussion

This study systematically delineates the knowledge structure and evolutionary trajectory of cell death research in LIRI by analyzing 2462 English-language publications from the WoSCC spanning 2005–2024. Conceptually, LIRI should not be regarded as a singular disease entity but rather as a convergent pathological endpoint manifesting across diverse clinical contexts. While its triggers, ischemic modalities, and reperfusion strategies vary according to disease background, bibliometric evidence consistently indicates that the activation, overlap, and dysregulation of multiple cell death modalities – including apoptosis, ferroptosis, and pyroptosis – constitute the central pathological foundation underlying LIRI onset and progression. Building upon this insight, the following discussion first addresses the overarching evolutionary characteristics of cell death research in LIRI before systematically examining the dominant death modalities and their potential translational implications across different clinical scenarios.

### Central role of cell death in LIRI

Keyword co-occurrence and temporal trend analyses reveal a clear mechanistic paradigm shift in LIRI research. Early investigations predominantly focused on ischemia–reperfusion–induced programmed apoptosis, with keywords such as apoptosis, caspase, and TNF-α maintaining central positions between 2005 and 2015. This trend reflects the prevailing framework of the time, wherein tissue injury was primarily interpreted through the lens of the inflammation–apoptosis axis. However, as the understanding of the regulatory networks governing cell death deepened, singular apoptotic models proved insufficient to explain the complex tissue damage and amplified inflammatory responses characteristic of LIRI.

From 2018 onward, multiple non-canonical forms of cell death have rapidly emerged at the forefront of research, notably ferroptosis, pyroptosis, and mitochondrial-associated death pathways, all demonstrating sustained surges in citation and keyword prominence. This evolution underscores that LIRI is unlikely to be driven by any single cell death mechanism; rather, it arises from the synergistic dysregulation of multiple death pathways in the context of oxidative stress, inflammatory signaling, and mitochondrial dysfunction^[^54–56^]^. Of particular note, terms such as mitochondrial dysfunction, reactive oxygen species, and lipid peroxidation frequently co-occur across studies of distinct death modalities, highlighting mitochondria as a central hub orchestrating the upstream triggers of diverse cell death processes^[^22,57^]^.

Consequently, at a mechanistic systems level, LIRI is best conceptualized as a dynamic, multi-pathway imbalance centered on mitochondrial injury and oxidative stress, wherein multiple cell death modalities collectively contribute. Within this framework, the heterogeneity observed across different clinical contexts of LIRI does not lie in the occurrence of cell death per se, but in which specific death modality predominates under particular pathological conditions and how it interacts with other concurrent death pathways.

### Lung transplantation–associated LIRI: ferroptosis and graft dysfunction

Among the various clinical contexts, lung transplantation–associated LIRI represents the most intensively studied and translationally promising domain. Cluster analyses reveal a strong coupling between lung transplantation and ferroptosis, while keyword timeline analyses indicate that PGD) has maintained sustained prominence from 2015 to 2024. The Sankey diagram further underscores that major research teams in the United States, Canada, and China consistently use lung transplantation models as their primary experimental framework, forming a relatively stable investigative trajectory.

From the perspective of cell death evolution, research in the lung transplantation field has shifted from early inflammation–apoptosis–centered injury models toward ferroptosis, characterized by lipid peroxidation and dysregulated iron homeostasis^[^5,58^]^. Notably, in 2022–2023, ferroptosis exhibited marked emergent strength and frequently co-occurred with terms such as rat model and EVLP, indicating that experimental investigations are increasingly adopting clinically relevant *ex vivo* organ platforms^[^59–61^]^. This translational emphasis is mirrored in the journal co-citation network, where American Journal of Respiratory and Critical Care Medicine and Transplantation occupy central nodes, reflecting the pressing clinical need for PGD prevention and graft protection strategies.

Mechanistically, LIRI following lung transplantation is distinguished by features such as high oxygen exposure, cold ischemia, and reperfusion in the context of lipid-rich substrates, all of which create a permissive environment for ferroptosis^[^62^]^. Consequently, ferroptosis may play a more dominant role than apoptosis or pyroptosis in this specific setting, providing a relatively clear target for therapeutic intervention.

### Cardiothoracic surgery–associated LIRI: mitochondrial regulation of apoptosis and autophagy

In the context of cardiothoracic surgery, particularly procedures involving cardiopulmonary bypass (CPB), LIRI typically manifests as a component of systemic ischemia–reperfusion responses^[^63^]^. Keyword co-occurrence analyses highlight myocardial infarction, heart failure, and reperfusion injury as closely interconnected, indicating that pulmonary injury is seldom studied in isolation but rather integrated within the broader framework of cardiovascular system damage.

Within the author co-citation network, researchers such as Hausenloy, Murphy, and Gottlieb have long focused on the RISK pathway, mitochondrial permeability transition pore (mPTP), and mitophagy, forming the theoretical nucleus of this research domain^[^64,65^]^. This pattern underscores that, in cardiothoracic surgical contexts, investigations of LIRI emphasize mitochondrial functional maintenance and its regulatory influence over apoptotic and necrotic thresholds. Accordingly, terms such as cardioprotection and lung protection are often discussed in tandem, reflecting efforts to achieve multi-organ protection through modulation of shared cell death pathways.

From the perspective of cell death modalities, the dynamic interplay between apoptosis and autophagy appears more pivotal than ferroptosis or pyroptosis in this setting. Mitophagy, in particular, is considered a protective mechanism that limits excessive apoptosis and sustains cell viability, whereas its dysregulation may exacerbate synchronized injury across cardiac and pulmonary tissues.

### Trauma- and shock-associated LIRI: inflammation-mediated pyroptosis

In contexts of severe trauma and hemorrhagic shock resuscitation, LIRI often occurs alongside systemic inflammatory response syndrome (SIRS)^[^66^]^. Although studies in this domain constitute a relatively small proportion of the overall literature, their mechanistic signatures are highly distinctive in keyword analyses. Notably, the NLRP3 inflammasome and pyroptosis emerged prominently after 2020 and frequently co-occur with inflammation-related terms such as neutrophil infiltration and IL-1β, indicating growing recognition of immune-driven cell death in this setting^[^57^]^.

Within the author network, research groups led by Jaeschke and Souza have focused on the coupling between innate immune activation and cell death pathways^[^67,68^]^. This subset of LIRI emphasizes microcirculatory dysfunction and immune cell–mediated tissue injury rather than purely hypoxia–reoxygenation effects. Consequently, gasdermin-D–mediated pyroptosis, characterized by strong pro-inflammatory features, may hold greater pathological relevance than apoptosis in trauma- and shock-associated LIRI^[^69^]^.

### Pulmonary embolism–reperfusion LIRI: unresolved cell death patterns

Notably, although reperfusion following pulmonary embolism (via thrombolysis or thrombectomy) involves a classic ischemia–reperfusion process, the 2462 articles included in this study did not reveal an independent keyword cluster centered on pulmonary embolism or thrombolysis. This indicates that this clinical scenario has not yet been systematically incorporated into the LIRI–cell death research framework.

From a cell death perspective, the dominant forms of cell death following reperfusion in pulmonary embolism remain unclear. This knowledge gap carries potential clinical significance, especially when balancing anticoagulation strategies with reperfusion injury mitigation. A deeper mechanistic understanding could inform novel intervention strategies.

### International collaboration and challenges in mechanistic integration

At the national and institutional level, LIRI–cell death research exhibits a clear Sino–US dual-core structure. North American teams lead in mechanistic innovation and clinical translation, whereas Chinese teams focus primarily on animal model development and intervention screening. However, when research is stratified by specific disease scenarios, clear collaboration gaps emerge, and a unified mechanistic framework across clinical models is lacking.

This disease- or procedure-specific fragmentation may limit the systematic integration of shared cell death mechanisms. For instance, whether ferroptosis broadly participates across different types of LIRI or is context-specific remains to be addressed through cross-scenario comparative studies.

### Methodological considerations, limitations, and future directions

This study has several limitations. First, data were sourced solely from the WoSCC, excluding other databases (e.g., PubMed, Scopus, or Chinese-language databases), which may not fully capture global research output. Second, the analysis was restricted to English-language publications, ensuring consistency but potentially underestimating contributions from non-English-speaking regions. Additionally, bibliometric analysis cannot replace mechanistic validation; the identified research hotspots require further experimental and clinical confirmation.

Considering the features across different clinical scenarios, future LIRI research urgently needs to shift from model-driven approaches toward an integrative framework centered on cell death phenotypes. Clarifying the dominant cell death modalities under varying etiologies, ischemia durations, and reperfusion strategies – and exploring their modifiability – could provide a robust theoretical foundation for precision prevention and intervention in LIRI.

## Conclusions

Research on cell death in LIRI has undergone a fundamental paradigm shift from a single “inflammation–apoptosis” model to a multi-pathway, coordinated dysregulation framework. Bibliometric evidence indicates that ferroptosis, pyroptosis, and mitochondria-related cell death pathways have rapidly emerged as frontier topics since 2018, with their dominance highly context-dependent: in lung transplantation, ferroptosis predominates due to high oxygen exposure and lipid peroxidation; in cardiothoracic surgery involving CPB, the dynamic balance between mitophagy and apoptosis determines the extent of cardiopulmonary co-injury; and in trauma or shock resuscitation, the NLRP3–pyroptosis axis drives immune-mediated tissue damage. Notably, classic ischemia–reperfusion scenarios, such as pulmonary embolism reperfusion, remain largely unexplored in the context of cell death, representing a clear knowledge gap. Current research is driven primarily by Sino–US dual cores, yet collaboration is largely confined within specific model systems, lacking cross-etiology and cross-organ mechanistic comparisons. Future studies should establish an integrative framework centered on “clinical scenario–cell death phenotype–intervention target” to advance LIRI research from descriptive mechanisms toward precise regulation.

## Data Availability

The original data presented in the study are included in the article and are available from the corresponding author upon reasonable request.

## Research registration unique identifying number (UIN)

This is a review, not applicable.
